# Indents

**Published:** 1905-03-15

**Authors:** 


### INDENTS.

**☞ Brief Articles of Technical or Scientific Value will receive notice and comment. Contributions invited.**

**Points About Vulcanization.** Dr. Christensen in this article gives some interesting facts about the vulcanization of rubber. The conclusion he arrives at after a number of experiments is that vulcanization, in order to produce the best results, should take place in *steam* only, at 320° F., and not in water at the same temperature. The purer the rubber is, the greater is the necessity of keeping the flask out of the water. In rubber containing a great deal of foreign material no particular difference is noticed. The following experiments are self-explanatory:

*Experiment* 1. 300 gm. water in the vulcanizer. Used two flasks, in each of which were placed blocks of rubber (eight thicknesses of ordinary rubber sheets)—in one, pure rubber, in the other, rubber mixed with foreign material. Time consumed to reach 320° F., twenty minutes; allowed to remain at that temperature one hour and forty-five minutes. Result: Pure rubber altogether porous; mixed rubber free from porosity.

*Experiment* 2. 200 gm. water in the vulcanizer. Blocks of the same size as before. Only pure rubber used in this and the following experiments. Time taken to reach 320° F., thirty minutes; vulcanized at that temperature one hour and forty-five minutes. Result: All blocks porous, but blocks in the upper flask less so than in the lower.

*Experiment* 3. 125 gm. of water. Lower flask empty; in upper flask the usual rubber blocks. Time and vulcanizing temperature same as before. Result: Perfectly free from porosity.

*Experiment* 4. 100 gm. of water. Blocks in both flasks.

Time and temperature the same. Result: Blocks in lower flask all porous; blocks in upper flask entirely free from porosity.—J. E. Heyke, D.D.S.

**Cardiac Syncope in the Course of Somnoform Anesthesia.** The writer begins his communication by remarking that the use of anesthetic mixtures containing ethyl and methyl chlorids and ethyl bromide is not wholly devoid of danger, and reports the case of a woman aged forty-eight, of weak constitution and with a past history of puerperal endometritis and neuralgic attacks of dental origin, who suffered from a severe cardiac syncope while under the influence of somnoform. While, as above stated, the patient was in a physical condition below par, yet a careful examination brought out no organic lesion—a negative result, as in the case of several previous examinations by other practitioners. The author emphasizes this point in order to show that the syncope which developed in the course of the anesthesia could not be accounted for in any way by the presence of any latent or active cardiac or pulmonary lesion.

The patient was at two different times placed under the influence of somnoform for the extraction of a large number of teeth. The first intervention was carried out without the slightest difficulty or discomfort. At the second intervention, sleep ensued shortly after the beginning of the inhalation and was accompanied by general contractions and opisthotonos. The extractions were performed in less than forty seconds, and were soon followed by a serious syncope, from which the patient recovered only after long and active stimulation of the respiratory and cardiac functions.

This case is reported mainly for the purpose of pointing out the fact that even the so-called innocuous anesthetics are not entirely devoid of danger. The same precautions as with chloroform and ether should be observed with the less dangerous anesthetics.—Dr. J. Dufour, in February *Cosmos*.

**Local Analgesia.** Barker describes his method of producing local anesthesia. He uses B-eucain, which is far less dangerous than cocain, while possessing analgesic properties little, if at all, inferior to it, and with the concurrent use of adrenalin for the purpose of securing a retardation of circulation equivalent to constriction of the part, he has removed some of the objections as to the duration of the analgesia, the extent of the area which can be dealt with and the amount of the toxic drug to be employed. It is necessary to keep within the safe dose of the drug, and to have at our disposal a large enough quantity of the fluid medium to render it possible to spread the analgesic agent over large areas. For ordinary surgical work Barker finds the following solution to answer well: Distilled water, 140 c.c.; B-eucain, 0.2 grams; sodium chlorid, 0.8 grams; 1 to 1,000 adrenalin chlorid solution, 10 minims. All this quantity of fluid can be used in an ordinary case if necessary and is quite sufficient for most. Twice as much may be injected without ill results. The duration of the insensibility is secured by the admitxure of the adrenalin. Without it sensation is only abolished by eucain for about fifteen minutes; with it, for three to four hours. But the analgesia is produced more slowly when adrenalin is employed with the eucain. It is therefore well before all larger operations to wait some thirty minutes after injection to allow time for the insensibility to become fully developed. After this the effect appears to deepen for a couple of hours. Waiting has another advantage. When eucain alone is employed the operation must be done at once. The tissues are still in a state of artificial edema which marks the anatomic details unpleasantly. By adding adrenalin to the eucain solution and waiting, the artificial edema has disappeared and details are very clearly seen. Rapid injection is to be avoided; sudden distention of the tissues is disagreeable, if not painful. The fluid should not be used cold nor too hot, for the same reason. All dragging on the parts is to be avoided, lest structures be pulled upon which lie beyond the area of infiltration. Barker has never seen any depressing effects follow the use of B-eucain in a long series of operations. A list of operations done under B-eucain analgesia is appended, and among these are the following: Abdominal sections, hernia operations, amputations, orchidectomy, removal of cyst of thyroid, removal of silver wire from around the patella, operations for fistula in ano-varicose veins, hydrocele, varicocele, etc.—*British Medical Journal.*

**Stovain: its Advantages and Incompatibility with Adrenalin.**

Stovain has over cocain two indisputable advantages: its toxicity is less than that of cocain, and its action upon inflamed tissue is decidedly beneficial. The fatal dose of stovain when injected into guinea-pigs is from fifteen to twenty centigrams; that of cocain is five centigrams. The proportionate toxicity of stovain to cocain is as 1:18.

The disadvantages of stovain are—first, the degree of anesthesia it induces is slightly less than that brought about with cocain, and is also of shorter duration; second, stovain is a pronounced vaso-dilator, and therefore the afflux of blood in the course of an operation is likely to interfere with what otherwise might have been a comparatively easy intervention. Some operators consider this vaso-dilator action decidedly advantageous, as thereby it may be possible to operate with the patient in the sitting position, there being no danger whatever of bringing about an anemia of the medulla with its consequent dangerous effects. But thus far it has not been shown that cocain syncope is due to bulbar anemia. The extreme vaso-dilator action of stovain cannot be modified by the addition of adrenalin, as the two agents are utterly incompatible. A stovain-adrenalin mixture produces in the guinea-pig the following three principal effects: first, marked vaso-constriction; second, diminution of the toxicity of stovain; third, it produces gangrene at the point of injection—a fact sufficiently potent to preclude its employment about the mouth.

The cocain-adrenalin mixture offers, on the other hand, all the advantages and none of the disadvantages of stovain, namely, perfect anesthesia in inflamed and non-inflamed tissues, weak toxicity, arterial hypertension and tonic action upon the heart, and local vaso-constriction. The author advocates the use of the following solution: 10 to 20 cc. of cocain solution 1:200, and 10 drops adrenalin 1,000.—Dr. Forsy, in January *Cosmos*.

**Splinting Loose Teeth.** Holding the teeth in firm position is a very great thing and should always be done for the treatment of pyorrhea, but of course the first thing to be done is the removal of the deposit; that must be done thoroughly—not as well as you can—you must do it thoroughly, because if a particle of deposit remains you will not get perfect tissue adhesion. The scraping of the roots in itself will produce a certain denudation of the alveolus, with concomitant growth of granulations. But my idea of the use of lactic acid is that it helps to produce the removal of the disorganized tissue that may remain, and to denude the surfaces not denuded by the instrument, and also to stop the openings of the canaliculæ—and we know there is no better germicide than lactic acid. But, moreover, I always insist upon my patients keeping the mouth thoroughly sterilized during the treatment, washing the mouth every hour or two with any sterilizing agent; otherwise the septic conditions remain in the mouth, and no wound will heal under such conditions. Asepsis must be thoroughly established. It is an error to think the gum the seat of disease; the gum is simply affected by the disease. The deposit is irritant, and if the tooth be in motion the irritation is much increased; but the gum will heal of itself when the deposit is removed; without medication it will become quite normal again. Keeping the teeth firm, once the deposit has been removed, is sufficient to allow the gums to heal; but this is aided by keeping the mouth in a thoroughly aseptic condition.

I saw an article in the *Medical News* in which Dr. Darby, of Philadelphia, was quoted as saying he had examined about 3,000 natives of Egypt—living, dead and mummies—and that in none of them had he seen pyorrhea, and very few that had decayed teeth. Now I have spent two seasons in Egypt, and, although I have not made such a thorough investigation, enough was shown me in my dragomen and boatmen and workmen, for me to feel convinced that the majority of them had pyorrhea; their inflamed gums and the rugged edges of the gums indicated it plainly.—Dr. Younger, in *Cosmos*.

**Remodeling of Ill-fitting Vulcanite Dentures.**

The method consists of flasking the case in such a way that it can be removed bodily from the flask for the purpose of removing the teeth from the vulcanite and putting them back in the flask, thereby getting rid of the old vulcanite altogether. An impression of the mouth is taken in the usual way, either in plaster or composition, and cast. The case is then tried on the cast. It may not go on at all, or be so wabbly that it will be difficult to discover what position is the correct one, but all one has to do is to cut away freely any excrescences or edges or rugæ that are in the way. If I have any difficulty in seeing what is keeping it, I wriggle the case slightly on the cast and cut it away at the white plaster marks that are left on the under surface. The great thing, I find, is (in the case of uppers) to get the palatal portion of the case lying as flat as possible, for if this be right, it is certain that the rest is, as it is not in the palate where changes occur in the mouth. All this can be done in a few minutes. The case is next waxed on to the cast and wax run into all the places to be filled up, but it is not necessary to have wax all the way under the case, as the edges are tight, to keep plaster from running in between it and the cast. Any teeth that require to be added are put on with wax, and any places where it is desirable to extend the case or add gum are waxed up. It may also be necessary to cover exposed pins, etc. The cast with the case on it is then placed in the lower part of the flask, and plaster brought up to the gum edge. The middle part of the flask is then lubricated inside with oil, vaselin or soap and placed in position. Plaster is then run round the faces of the teeth and gum and crowns of the teeth. The plaster is then trimmed up, smoothed and lubricated, the palatal portion which remains filled in with plaster, and the top of the flask put on. When the plaster has set and the flask has been opened, the case will be found embedded in a ring of plaster in the middle part of the flask; this having been well lubricated allows the plaster with the case to be removed. The ring of plaster is then broken into three or four sections and the case liberated. These sections of plaster are now put back into the middle part of the flask where they were before. The top part of the flask is then put on, and the lower part with the cast used as the plug; or it can be packed the other way if desired. The teeth are then removed from the case and fitted into their places in the plaster. If the back teeth are diatoric and the vulcanite will not come out of the holes, I have found that a very good way is to place them in a crown or inlay furnace and burn it out. I heat them until they are red hot, when they will be found nice and clean.—T. Evans Johnston, *Dental Record.*

**How to Keep the Teeth.** Prevention is easier and cheaper than cure. Especially is this true as regards the preservation of the teeth. Decay of the teeth is the result of either ignorance or neglect in practically every instance.

If people clearly realize the importance of keeping the mouth and teeth in a hygienic condition, it would save them both money and pain. Indigestion, anemia, auto-intoxication and allied conditions would be more amenable to treatment, for a septic state of the mouth undoubtedly infects the system to a greater or less extent.

Few people understand how foul and repulsive a place the mouth may become, and often is, if neglected. Perfect health is rare. The majority of people have some weak or diseased part which affects the secretions, and particularly those of the mouth. Sordes is deposited on the teeth, and fills the crypts and lacunæ in the mucous membrane of this cavity; the tongue is coated to a certain extent.

If there is any catarrh of the nose and throat, or an established habit of breathing through the mouth, the conditions are made still worse. The breath is charged with the odors resulting from vitiated secretions and highly offensive.

If these deposits are not removed, they are swallowed or absorbed, injuring all the tissues with which they come in contact.

No teeth, however hardy, can resist such an environment. Sometimes the disease set up extends from the teeth to the alveolar processes, the jaw-bone is destroyed, or even worse damage done.

The teeth, nowadays, do not get the exercise they did in less civilized times in masticating tough foods. The cook does a part of this work for mankind to-day. As a result, the teeth are less strong and resistant, requiring to be artificially cared for.

Sweetmeats and starchy foods induce rapid fermentation, and predispose the teeth to decay. A diet containing fresh fruit, vegetables, meat in reason, and corn bread, is excellent to preserve the teeth. Eat slowly and chew the food well. Adherent particles should be removed after every meal, the teeth brushed, and the mouth carefully rinsed out with a solution of listerine, the standard antiseptic.

Once a month the teeth should be cleaned well with pumice stone, and this application *is preferably made* by the dentist. But with a little patience any one can learn to do it. Sit down before a shaving mirror to perform the operation. A short, slender stick with a small disc of hard rubber fitted to it, makes an admirable cleaning instrument. Moisten same, dip into the powdered pumice stone, and rub up and down, and into every crevice. Rinse well with listerine solution.

The person who carefully follows these instructions, as given to him by his physician, can, other things being equal, count on preserving his teeth in a useful state to an advanced age, and will enjoy better general health.—*Medical Brief.*

**The Downfall of Therapeutics.** The following editorial from *American Medicine* shows clearly the present status of therapeutics and materia medica: "Before the development of the natural sciences placed medicine as an art among the applied sciences, therapeutics was the most important branch of medical study and practice. Among the medical sects the same observation holds true at the present time. During the last half century medical progress however, the pursuit of exact medical knowledge, has led students chiefly into surgery, diagnosis, pathology and bacteriology. Definite knowledge of the intimate reactions of the metabolic processes of our bodies with foreign agents introduced as drugs is just coming into sight with the growth of the new science of pharmacology. and also necessarily therapeutics at present lags behind. All of which is new to no one who sees, and is introduced simply to draw attention to an unfortunate practical result of this state of affairs. For some years progress in materia medica and therapeutics has seemed almost to lie in the hands of lay manufacturers. With their new compounds and their disguised old ones, the drug makers have kept the materia medica expanding at an alarming rate. This condition is by no means wholly to be deplored, as the pharmaceutists have contributed much to the comfort of physician and patient. However, the tremendous preponderance of commercialism in recent therepeutic progress has brought with it some ill conditions. Money-making attracts the unscrupulous, and the medical profession has difficulty in separating the wheat from the tares. Any good new laboratory product of therapeutic value is immediately imitated or attacked. There develops intense feeling between foreign and domestic manufacturers, and all the while the poor doctor, as the bone of contention, is overwhelmed with 'literature' and medical journals (*sic*) and agents. Contradictions multiply until mercury and quinin appear to be about the only agents of whose qualities the physician may feel reasonably sure! The prize—the patronage of the profession—being of great commercial value, many subterfuges are employed by some to enable them to share in it. Official positions in medical bodies at times are sought, and at other times are appropriated by not too nice strategy. Medical journals are subsidized and new ones are published. A number of makers pay a regular stipend secretly to complaisant doctors here and there over the country, who, in return, are expected to read 'useful' papers and at every possible turn to uphold the wares of the benefactor. Thus it comes that therapeutics has reached its present low estate. Scientific physicians give their time to diagnosis and pathology, and limit themselves to simplest measures of treatment. Others make a diagnosis and then choose the remedy that the maker says is best for that condition. Many among us who love the study of means to alleviate suffering have endeavored faithfully to recreate professional interest in pure therapeutics, but the combat with intrenched wealth and monopoly is an unequal one in the face of general professional lethargy. But hope is not to be abandoned."

**Working Porcelain.** As to the thickness of the platinum, that is always a matter of judgment. In the cap or band it may be made of great thinness, far below No. 30 gauge. For plates, from No. 30 to No. 32 gauge is most frequently employed in my office; but it is always a matter of judgment and discretion.

As to cooling off rapidly or slowly, that is also a matter of judgment. In an inlay it is a matter of no moment if in the first three bakings it is cooled off with great rapidity. At the last baking, however, it is desirable to cool it off more slowly. I had always believed and been taught to believe, from the beginning, that one must exercise extreme care to prevent artificial teeth from cracking after one had exposed them to the heat of soldering, or to the heat necessary with porcelain prosthetic work. After having succeeded in obtaining—in the small quantities of laboratory experiment—what I have called prosthetic porcelain, it took me two years of constant labor to produce this material in a sufficient quantity for mercantile purposes. It was during that time that I had occasion to try innumerable experiments in attaching artificial teeth to platinum bases, under all sorts and conditions I believe out of many hundreds of such experiments there were only two where the artificial teeth cracked, when removing the piece at a white heat out into the open air of the laboratory. That can be done with prosthetic porcelain perfectly well. It is so tough and elastic that it will not itself crack by this sudden change of temperature into the free air. If you throw cold water on it, it will of course crack. Artificial teeth under those circumstances crack with extreme frequency in the free air. It shrinks? Of course it does. You can not get solidity in any other way. Any porcelain powder which does not shrink does not become solid. But it can not be driven. It is a feminine material.

If you pack it gently into its position and repack it, always under the same conditions, you will eventually get a perfectly solid and reliable body. It can be melted and re-melted a great number of times without the least degree impairing its qualities. That is of value, especially if an accident should occur; then it is possible in a case of continuous-gum work to repair it by repeated meltings without danger of injuring the original material.

Of course, everything relating to this class of work requires experience. It requires judgment, it requires self-control; but there is no such thing as a really scientific and skillful dentist who can not, if he choose to give himself the trouble, produce better results with porcelain than he ever succeeded in getting with gold. About that there is no question in my mind; but I am very glad there exists such a degree of conservatism in the profession as to lead it to continue steadfast in the employment of the materials to which it is accustomed. If we were to attempt immediately to attain to the full efficiency of every important discovery, we should make a mess of it. It is better that we go slowly, and that we reach our goal through the process of natural evolution.—N. S. Jenkins, in *Cosmos*.

**Cresylone.** Cresylone is a non-toxic, permanent preparation, and inasmuch as it will make a clear solution with water in all proportions it is superior to other cresols and to carbolic acid as a germicide and disinfectant. Its germicidal and therapeutic properties are due to cresylic acid, of which it contains fifty percent. To the surgeon it commends itself as a general disinfectant for the hands and instruments. For this purpose a two percent solution should be employed. As it is easier of application than soap and disinfectant fluids, and thoroughly removes dirt, fatty matter, etc., from the skin, it may be used to advantage in rendering the field of operation aseptic before a surgical procedure. It does not injure metallic and rubber instruments, but celluloid articles are said to become friable and useless under its action.

As it renders the operator's hands soft and flexible, cresylone accentuates the *tactus eruditus* upon which the success of the surgeon so often depends.

The value of cresylone as an antiseptic has been confirmed by clinical experience. Although its antiseptic power is marked, it is free from irritating properties when applied in a solution of the proper strength. For the treatment of wounds a one to two percent solution should be employed. This is best applied in spray form and will promptly inhibit sepsis and promote granulation. A weak solution may also be of service in obstetrical practice, and prove useful as a wash in major and minor gynecological operations.

Cresylone will completely arrest the development of pus organisms, and is, therefore, indicated in the various suppurations with which the general practitioner has to contend. In the treatment of otorrhea, irrigation with a half percent solution is said to be of benefit. A solution of the same strength may also be of value in the treatment of ozena.

As it removes the odor, it may prove of service in cases of gangrene in which an operation is out of the question. Even during an operation it will render life bearable to the surgeon performing amputation, in which event cloths saturated with a strong solution of cresylone should be applied to the limb about to be removed. In cancer of the cervix uteri the application of gauze saturated with a solution of cresylone will effectually remove the dreadful odor that accompanies this disease. For disinfecting sputa and stools cresylone commends itself in the sick-room, hospital-ward, schools, prisons, etc., although kreso will probably be preferred for this purpose on the score of economy.

Therapeutically, the use of cresylone has been suggested in various indications, and is supposed to be of benefit in the treatment of gonorrhea, lupus, tonsillitis, eczema and cystitis of the female. For washing out the bladder or any other viscus, however, a weak solution, not over half percent in strength, should be employed. In the treatment of dysentery, enemata of a pint and a half percent solution, thrice daily, may effect a cure. But in this connection it may be well to bear in mind that while all the cresol preparations (to which cresylone belongs), are comparatively safe, they are not altogether harmless.

Cresylone is marketed in pint bottles only.

